# Preliminary Cellular and Contamination-Related Observations From Adinizer-Processed Microfragmented Adipose Tissue: A Technical Note on Three Donors

**DOI:** 10.7759/cureus.115366

**Published:** 2026-08-28

**Authors:** Yonghyun Yoon, Jihyo Hwang, Jungyoun Kim, Teinny Suryadi, Anwar Suhaimi, Jeimylo C de Castro, King Hei Stanley Lam

**Affiliations:** 1 Orthopedics, International Academy of Musculoskeletal Medicine, Hong Kong, HKG; 2 Orthopedics, International Academy of Regenerative Medicine, Incheon, KOR; 3 Orthopedic Surgery, Hallym University Kangnam Sacred Heart Hospital, Seoul, KOR; 4 Orthopedic Surgery, Incheon Terminal Orthopedic Surgery Clinic, Incheon, KOR; 5 Physical Medicine and Rehabilitation, Medistra Hospital, Jakarta, IDN; 6 Physical Medicine and Rehabilitation, Synergy Clinic, Jakarta, IDN; 7 Physical Medicine and Rehabilitation, Hermina Podomoro Hospital, Jakarta, IDN; 8 Rehabilitation Medicine, University Malaya Medical Centre, Kuala Lumpur, MYS; 9 Rehabilitation Medicine, University Malaya, Kuala Lumpur, MYS; 10 Research, Adventist University of the Philippines, Cavite, PHL; 11 Clinical Research, International Academy of Regenerative Medicine, Incheon, KOR; 12 The Board of Clinical Research, The Indonesian Malaysian Regenerative Institute, Jakarta, IDN; 13 The Board of Clinical Research, The International Association of Musculoskeletal Medicine, Kowloon, HKG; 14 Faculty of Medicine, The Chinese University of Hong Kong, New Territories, HKG; 15 Faculty of Medicine, The University of Hong Kong, Hong Kong, HKG; 16 The Board of Clinical Research, The Hong Kong Institute of Musculoskeletal Medicine, Kowloon, HKG

**Keywords:** adinizer, adipose-derived stromal cells, cell viability, endotoxin, flow cytometry, microfragmented adipose tissue, mycoplasma, regenerative medicine

## Abstract

Background

Microfragmented adipose tissue (MFAT) is a mechanically processed adipose-tissue preparation that retains extracellular matrix and heterogeneous stromal and immune cell populations. Because intact MFAT is not a single-cell suspension, cellular enumeration requires laboratory dissociation. This retrospective technical note describes preliminary cellular observations and limited contamination screening results in Adinizer-processed MFAT from three donors.

Methods

De-identified laboratory records from three female donors were retrospectively reviewed with institutional review board approval and written informed consent. Residual lipoaspirate was mechanically processed using serial Adinizer cutting units (2400, 1200, 600, and 400 μm; five passes at each size), centrifuged at 1,200 × g for 5 minutes, cleared of the upper oil-containing layer, and passed through a final 200-μm cutting unit. A 3-mL aliquot of refined MFAT was enzymatically dissociated. Cell concentration and viability were measured in duplicate as technical replicates and averaged. Cells were stained for CD34, CD45, CD90, and CD146. Mycoplasma and endotoxin findings were reviewed as limited contamination screening results.

Results

Donor ages ranged from 43 to 70 years and BMI from 34.5 to 39.9 kg/m². Mean recovered cell concentrations ranged from 1.41 × 10⁵ to 8.41 × 10⁵ cells/mL, corresponding to 2.82 × 10⁵ to 1.68 × 10⁶ total recovered cells from each 3-mL aliquot. Mean viability ranged from 65.8% to 78.6%, with calculated viable recovered cell counts of 2.11 × 10⁵ to 1.32 × 10⁶. Laboratory-reported ASC-like fractions ranged from 15.4% to 24.4%, and leukocyte fractions from 22.6% to 32.0%. Mycoplasma testing was negative in all samples, and all endotoxin results met the laboratory acceptance criterion of <1 EU/mL.

Conclusions

Viable cells, including a laboratory-defined ASC-like fraction, were recovered after enzymatic liberation of Adinizer-processed MFAT. Recovered cell yield varied substantially among donors, with no consistent monotonic pattern across the obese BMI range represented. The ASC-like fraction suggests preservation of a stromal/progenitor-compatible cellular component after mechanical processing; however, the limited marker panel and absence of functional assays preclude conclusions regarding definitive mesenchymal stromal/stem-cell identity or functional stemness. Mycoplasma and endotoxin findings should be interpreted only as limited contamination screening results, not as evidence of sterility or product safety. Larger prospective studies with complete flow-cytometric documentation, broader marker panels, and functional assays are required.

## Introduction

Adipose tissue has become an important source of orthobiologic material because it contains extracellular matrix, perivascular elements, immune cells, endothelial-lineage cells, and adipose-derived stromal/progenitor cells. Microfragmented adipose tissue (MFAT) is prepared by mechanically resizing adipose tissue into smaller injectable fragments while retaining a native tissue scaffold. Unlike culture-expanded cell products, MFAT is generally used as a minimally manipulated tissue-based preparation in point-of-care regenerative medicine applications.

Several MFAT systems have been evaluated for cellular composition, and prior studies have shown that cell recovery and stromal-cell proportions may vary according to the processing device and analytical method [1,2]. Related studies of adipose-derived stromal vascular fraction have also demonstrated that recovered cell yield and viability can vary with donor characteristics, tissue source, and processing conditions [3,4]. This device- and method-dependence is clinically relevant because intact MFAT is a tissue product rather than a single-cell suspension; direct cell counting is therefore not feasible without additional dissociation. Enzymatic liberation of cells from MFAT is not part of routine point-of-care MFAT preparation, but it can be used as a laboratory method to characterize the cellular component of the tissue product.

The Adinizer (BSL Co., Ltd., Busan, Republic of Korea) is a blade-based mechanical processing system designed to produce injectable, mechanically processed adipose tissue without enzymatic digestion [5,6]. Previously published Cureus case reports from this group have described clinical applications of Adinizer-processed MFAT in osteonecrosis of the femoral head, degenerative lumbar spondylolisthesis, disc-driven cervical myelopathy, and knee osteoarthritis [7-10], but basic cellular characterization of the resulting product has not been reported by this group. Therefore, this technical note describes preliminary observations of recovered cell yield, viability, a limited laboratory-reported flow-cytometric marker pattern, and mycoplasma/endotoxin contamination screening results following analytical enzymatic cell liberation from three de-identified residual adipose tissue specimens processed with the Adinizer system. Enzymatic liberation was performed solely for laboratory characterization and was not part of the clinical MFAT preparation. We also descriptively examined donor-level variation in recovered cell yield across the obese BMI range represented by these three donors. These observations are descriptive and hypothesis-generating and were not intended to establish definitive mesenchymal stromal/stem-cell identity, cellular potency, clinical efficacy, device superiority, sterility, clinical safety, or a generalizable relationship between BMI and cellular yield.

## Materials and methods

Study design and ethics

**Figure 1:**
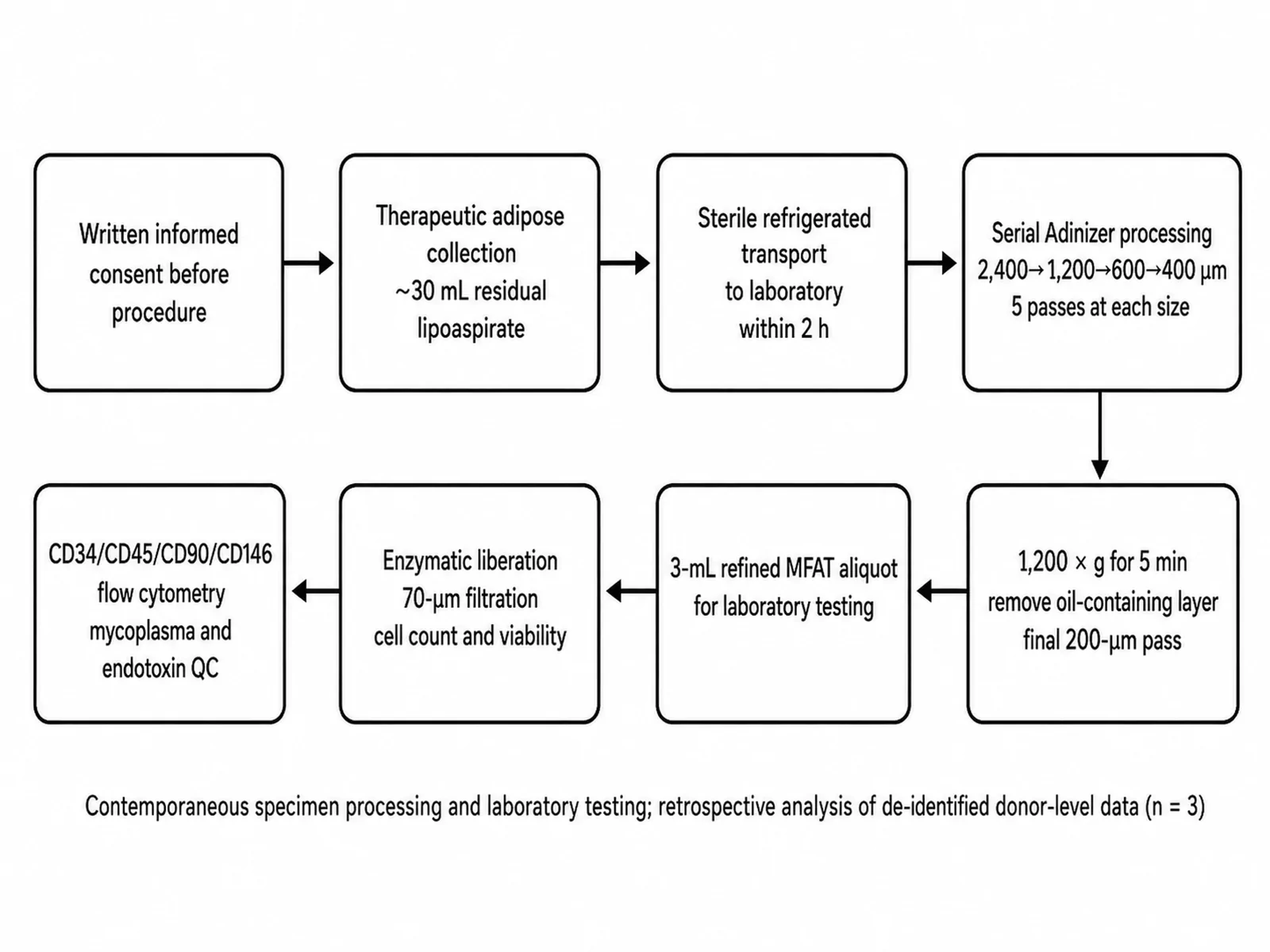
Workflow for retrospective characterization of Adinizer-processed MFAT. Residual lipoaspirate obtained during therapeutic procedures was transported under refrigerated sterile conditions and processed using serial Adinizer cutting blades (2,400 → 1,200 → 600 → 400 μm; five passes at each size), followed by centrifugation at 1,200 × g for 5 minutes, removal of the oil-containing layer, and a final 200-μm pass. A 3-mL refined MFAT aliquot from each donor then underwent enzymatic liberation, 70-μm filtration, cell counting, viability assessment, flow cytometry (CD34/CD45/CD90/CD146), and mycoplasma/endotoxin contamination screening. MFAT: microfragmented adipose tissue.

This technical note retrospectively reviewed de-identified donor-level laboratory data generated from residual adipose tissue specimens obtained during therapeutic procedures. The overall workflow, including written consent, specimen acquisition, Adinizer processing, laboratory testing, and retrospective donor-level review, is summarized in Figure 1. The protocol was approved by the Hallym University Kangnam Sacred Heart Hospital Institutional Review Board (HKS IRB 2026-06-023-002). All three participants received an explanation of the research and provided written informed consent before the therapeutic procedure; consent was not waived. Residual specimens were collected, transported, processed, and analyzed on the day of the procedure. Direct identifiers were removed before retrospective analysis.

Specimen source and donor characteristics

Three female donors were included. Approximately 30 mL of residual lipoaspirate was available from each donor after removal of the lower tumescent-fluid and blood layer. Each specimen was immediately placed in a sterile refrigerated transport container, transferred to the cell-analysis laboratory within 2 hours, and processed on receipt without additional storage. Anthropometric data were obtained from body-composition reports performed on the same date as specimen processing. Donor identifiers were removed before analysis. BMI, body fat percentage, resting metabolic rate, and relative skeletal muscle index were recorded when available.

MFAT preparation and enzymatic cell liberation

Residual lipoaspirate was processed with the Adinizer cutting-blade system (BSL Co., Ltd., Busan, Republic of Korea). Each sample was passed sequentially through 2,400-, 1,200-, 600-, and 400-μm cutting units, with five passes at each size, to prepare approximately 10 mL of mechanically processed MFAT. The product was centrifuged in a swing-bucket rotor at 1,200 × g for 5 minutes. The processing record described four visually distinct layers as mechanical stromal vascular fraction, extracellular matrix, adipose tissue, and oil. The upper oil layer and approximately 3 mL of the immediately underlying oil-containing adipose layer were removed. The remaining product was passed through a final 200-μm Adinizer cutting unit to prepare approximately 7 mL of refined MFAT. In this technical note, refined MFAT denotes this final mechanically processed product. The tissue underwent repeated passage through the Adinizer cutting units during mechanical processing, as described above. No additional homogenization, agitation, or mixing was performed after the final 200-μm passage before sampling. A 3-mL aliquot was then obtained from the final refined MFAT preparation immediately after completion of mechanical processing and loaded into a prefilled syringe for laboratory analysis.

For analytical cell liberation, 3 mL of refined MFAT was mixed with 1 mL of NB6 solution (3 mg/mL; Nordmark Biochemicals, Uetersen, Germany; catalog no. N0002779) and incubated at 37°C with agitation at 100-150 rpm for 1 hour. The reaction was stopped by adding 4 mL of 2% Helios human platelet lysate (AventaCell BioMedical, Atlanta, GA, USA) in phosphate-buffered saline (PBS). Samples were centrifuged at 500 × g for 3 minutes, the supernatant was removed, and the pellet was resuspended in 1 mL of 2% human platelet lysate/PBS. The suspension was passed through a 70-μm strainer, followed by a further 1-mL wash through the strainer, resulting in an approximately 2-mL recovered-cell suspension. Enzymatic treatment was performed solely for laboratory characterization and was not part of the clinical MFAT preparation.

Cell counting and viability assessment

After enzymatic digestion and filtration, recovered cell concentration and viability were measured using a LUNA-FL automated fluorescence cell counter (Logos Biosystems, Anyang, South Korea). Cell counting and viability assessment were performed in duplicate as technical replicate measurements from the same analytical preparation and run for each donor sample, and the mean of the two measurements was used for analysis. These duplicate measurements did not represent independent MFAT processing runs. Total recovered cells from each 3-mL MFAT aliquot were calculated from the mean measured cell concentration and the approximately 2-mL post-filtration suspension volume. Calculated viable recovered cells were obtained by multiplying the total recovered cell count by the mean viability fraction.

Flow cytometry

Before flow cytometry, red blood cell lysis was performed. A total of 5.0 × 10⁵ recovered cells were transferred into a conical tube, PBS was added to reach 1 mL, and 9 mL of red blood cell lysis buffer was added. After a 10-minute room-temperature incubation, the sample was centrifuged at 500 × g for 3 minutes, washed once in 2% human platelet lysate/PBS, and resuspended in 0.5 mL. For staining, 200 μL of cell suspension per well was combined with 1 μL each of FITC anti-human CD34 antibody (catalog 343504), Brilliant Violet 510™ anti-human CD45 antibody (catalog 368526), APC/Cyanine7 anti-human CD90 (Thy1) antibody (catalog 328132), and APC anti-human CD146 antibody (catalog 361016) (BioLegend, San Diego, CA, USA). Samples were incubated at 4°C for 30 minutes, centrifuged at 500 × g for 3 minutes, resuspended in 100 μL of 2% human platelet lysate/PBS, and analyzed using a CytoFLEX flow cytometer (Beckman Coulter, Brea, CA, USA). The laboratory-provided analytical scheme used forward/side scatter followed by CD45/CD34 and CD90/CD146 classification to report leukocyte and adipose-derived stromal/progenitor-compatible fractions. Because the retrospective compiled report did not include raw FCS files, event-level plots, exact gate thresholds, denominator definitions, compensation matrices, staining controls, viable-cell gating details, or isotype/FMO controls, the reported fractions represent laboratory-generated summary classifications. They cannot be independently reproduced or reanalyzed from the available dataset. Therefore, the reported stromal/progenitor category is described conservatively as an ASC-like fraction. The endothelial-cell category was not reportable. Additional markers such as CD73, CD105, CD31, CD235a, and HLA-DR, as well as culture-adherence and differentiation assays, were not available.

Mycoplasma and endotoxin testing

Mycoplasma and endotoxin testing was performed by the testing laboratory as limited contamination screening assessments. Mycoplasma results were reported qualitatively as negative or positive. Endotoxin results were reported as acceptable or unacceptable using a laboratory acceptance criterion of <1 EU/mL. The compiled source report did not include the assay platform, manufacturer, kit information, analytical sensitivity, positive/negative control details, or sample-interference testing information. Accordingly, these results were treated as limited laboratory-reported contamination screening outcomes rather than comprehensive quality-control release data.

Statistical analysis

Because only three donors were included in this descriptive report, no inferential statistical testing was performed. Data are presented descriptively at the donor level. The relationship between BMI and recovered cell yield was assessed descriptively by tabular summary and scatter plotting; no correlation coefficient, regression model, or trend line was calculated.

## Results

Donor characteristics

The three donors were female, with ages of 46, 43, and 70 years. BMI values were 39.9, 36.2, and 34.5 kg/m², respectively. Body fat percentage ranged from 45.7% to 51.6%. Donor-level anthropometric characteristics are summarized in Table 1.

**Table 1:** De-identified donor characteristics. BMI: body mass index; RMR: resting metabolic rate; RSMI: relative skeletal muscle index; WHO: World Health Organization.

Variable	Donor 1	Donor 2	Donor 3
Age (years)	46	43	70
Sex	Female	Female	Female
Height (cm)	153.1	147.3	150.5
Weight (kg)	93.5	78.6	78.1
BMI (kg/m²)	39.9	36.2	34.5
WHO BMI category	Obese	Obese	Obese
Body fat (%)	45.7	51.6	48.0
RMR (kcal/day)	1,430	1,175	1,221
RSMI (kg/m²)	7.63	7.12	7.91

Cell count, viability, and flow cytometry

Donor-level cellular observations are summarized in Table 2. Mean recovered cell concentrations from duplicate technical replicate measurements performed within the same analytical run ranged from 1.41 × 10⁵ to 8.41 × 10⁵ cells/mL. Total recovered cell counts calculated from these mean concentrations for the 3-mL refined MFAT aliquots ranged from 2.82 × 10⁵ to 1.68 × 10⁶ cells. Mean viability ranged from 65.8% to 78.6%, corresponding to calculated viable recovered cell counts of 2.11 × 10⁵ to 1.32 × 10⁶. Laboratory-reported leukocyte fractions ranged from 22.6% to 32.0%, and ASC-like fractions ranged from 15.4% to 24.4%. Mycoplasma testing was reported as negative in all three samples, and all endotoxin results met the testing laboratory's acceptance criterion of <1 EU/mL.

**Table 2:** Recovered cell concentration, viability, laboratory-reported flow-cytometry fractions, and limited contamination screening results. ASC-like: adipose-derived stromal/progenitor-compatible; EU: endotoxin unit; MFAT: microfragmented adipose tissue. Recovered cell concentration and viability values represent the mean of duplicate technical replicate measurements performed within the same analytical run for each donor. Total and viable cell counts were calculated from these mean values after enzymatic liberation from a 3-mL MFAT aliquot and should not be interpreted as the intact-tissue cellular content or a clinical cell dose.

Variable	Donor 1	Donor 2	Donor 3
Recovered cell concentration (cells/mL)	1.41 × 10⁵	8.41 × 10⁵	6.73 × 10⁵
Total recovered cells from 3-mL MFAT aliquot	2.82 × 10⁵	1.68 × 10⁶	1.35 × 10⁶
Viability (%)	74.7	78.6	65.8
Calculated viable recovered cells	2.11 × 10⁵	1.32 × 10⁶	8.88 × 10⁵
Leukocyte fraction (%)	30.0	32.0	22.6
ASC-like fraction (%)	19.1	24.4	15.4
Mycoplasma test	Negative	Negative	Negative
Endotoxin test	<1 EU/mL; acceptable	<1 EU/mL; acceptable	<1 EU/mL; acceptable

BMI and cellular yield

**Figure 2:**
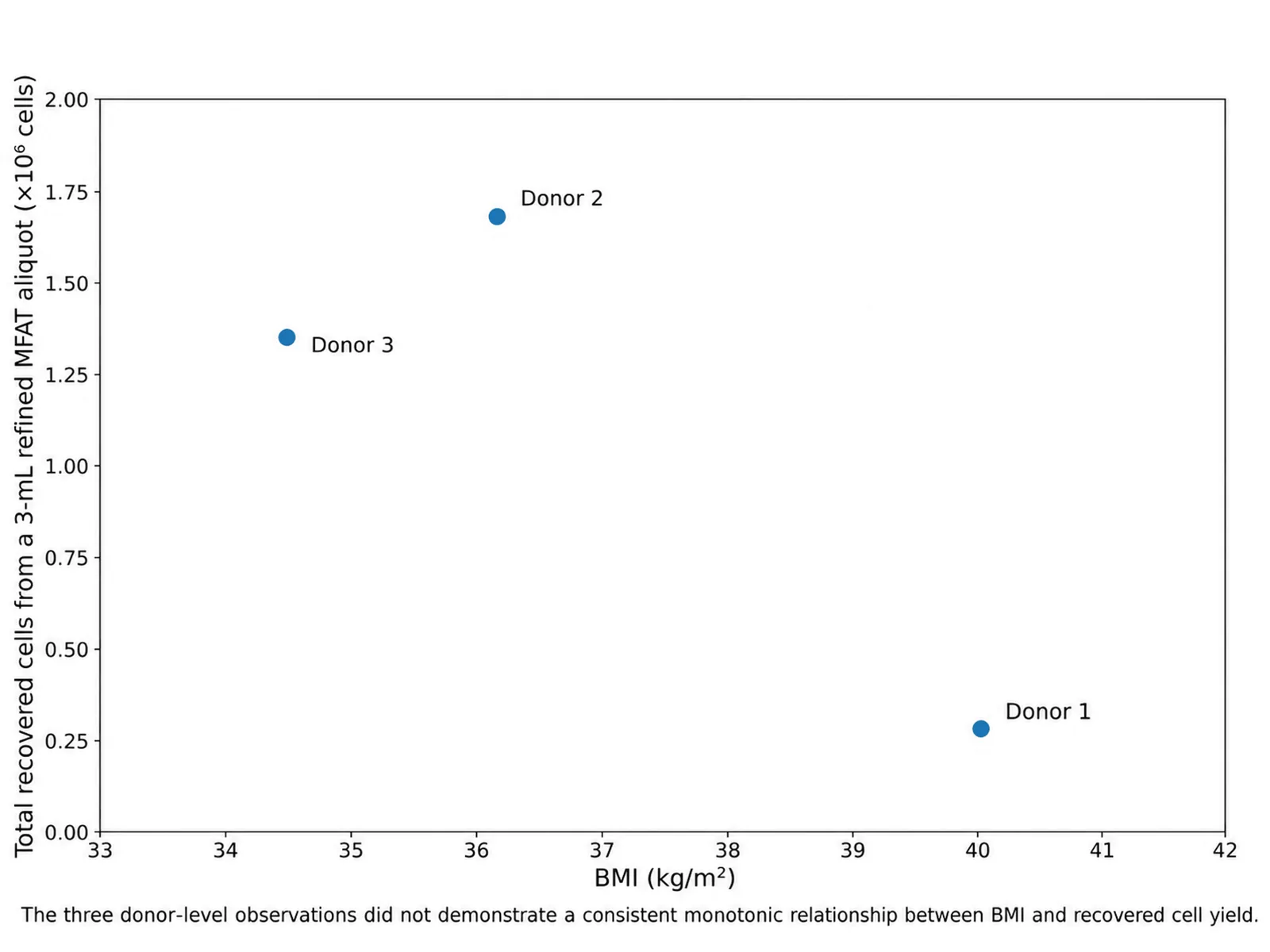
BMI and recovered cell yield after enzymatic liberation. Scatter plot showing donor BMI and total recovered cell counts from a 3-mL refined MFAT aliquot. All three donors had BMI values within the obese range. In these three descriptive donor-level observations, no consistent monotonic pattern between BMI and recovered cell yield was observed. BMI: body mass index; MFAT: microfragmented adipose tissue.

The descriptive relationship between BMI and recovered cell yield is illustrated in Figure 2. All three donors had BMI values within the obese range. The highest total recovered cell count was observed in Donor 2 (BMI 36.2 kg/m²), whereas Donor 1, who had the highest BMI (39.9 kg/m²), showed the lowest recovered cell count. Donor 3, who had the lowest BMI (34.5 kg/m²), showed an intermediate recovered cell count. Within this restricted obese BMI range, the three donor-level observations did not demonstrate a consistent monotonic pattern between BMI and recovered cell yield.

## Discussion

This retrospective technical note provides preliminary cellular observations and limited contamination screening results for Adinizer-processed MFAT using de-identified laboratory data from three residual adipose tissue specimens. The standardized mechanical protocol was followed by enzymatic liberation solely for analytical purposes, enabling descriptive quantification of recovered nucleated cells. Recovered cell counts varied markedly among donors, and the three observations did not show a consistent monotonic pattern with BMI. The available laboratory-reported marker panel supported an ASC-like classification but did not satisfy complete criteria for defining mesenchymal stromal/stem cells. All samples were reported as negative for mycoplasma and met the testing laboratory's endotoxin acceptance criterion of <1 EU/mL; these findings are interpreted only as limited contamination screening results.

Interpretation of BMI-related findings

BMI is a clinically convenient measure of body habitus, but it does not directly characterize adipose tissue cellularity, vascularity, fibrosis, inflammatory composition, harvest-site quality, or processing recovery. Previous studies of adipose-derived stromal vascular fraction have reported associations of recovered cell yield with donor-related and procedural factors, including age, BMI, lipoaspirate volume, and tissue source, although the magnitude and direction of these associations are not uniform across studies [3,4]. In the present three-donor dataset, all donors had BMI values within the obese range, and recovered cell yield varied substantially. The descriptive pattern shown in Figure 2 did not demonstrate a consistent monotonic relationship between BMI and recovered cell yield within this restricted BMI range. These observations cannot be extrapolated to normal-weight or overweight individuals and do not permit assessment of BMI-related differences across conventional weight categories. Given the extremely small sample size, these observations should be interpreted as preliminary and hypothesis-generating only.

Interpretation of CD marker findings

The terminology used to describe adipose-derived cell populations is critical. According to the International Society for Cellular Therapy minimal criteria and its subsequent nomenclature update, mesenchymal stromal/stem cells should demonstrate plastic adherence, expression of CD105, CD73, and CD90, lack of specified hematopoietic and immune markers, and trilineage differentiation capacity [11,12]. The present retrospective dataset included only CD34, CD45, CD90, and CD146 summary results. It did not include CD73, CD105, CD31, CD235a, HLA-DR, raw flow-cytometry files, independently reproducible gating information, culture adherence, or osteogenic, adipogenic, and chondrogenic differentiation assays. Therefore, the laboratory-reported population should be described as an ASC-like fraction rather than definitive mesenchymal stromal/stem cells.

This conservative terminology is especially important for uncultured adipose-derived preparations. In stromal vascular fraction and adipose-derived stromal cell nomenclature, CD34 positivity can be compatible with uncultured adipose-derived stromal/progenitor populations, whereas culture-expanded MSC-like cells are generally characterized using a different marker profile [13]. Multicolor flow-cytometric studies of stromal vascular fractions obtained from lipoaspirate and microfragmented adipose counterparts have used broader panels, including CD31, CD34, CD45, CD73, CD90, CD105, and CD146, to distinguish heterogeneous stromal, perivascular, endothelial, and hematopoietic populations [14,15]. The percentages reported here reflect the categories assigned by the testing laboratory rather than an independently reanalyzed phenotype. Nevertheless, the consistent recovery of a laboratory-defined ASC-like fraction across all three donor samples suggests that a stromal/progenitor-compatible cellular component remained recoverable after Adinizer mechanical processing. These observations should be regarded as preliminary product characterization rather than definitive evidence of fully characterized mesenchymal stromal/stem cells.

Safety-related findings

Mycoplasma testing was reported as negative in all samples, and all endotoxin results met the testing laboratory's predefined acceptance criterion of <1 EU/mL. In clinical-grade cell-therapy manufacturing, microbiological quality-control frameworks commonly include sterility testing together with mycoplasma and endotoxin assessment as complementary safety measures [16,17]. The present results are more limited and should not be interpreted as equivalent to a comprehensive manufacturing-release program. They do not establish sterility, absence of all microbial contamination, product potency, or clinical safety. Interpretation is further limited because the compiled report did not include the assay platform, kit, analytical sensitivity, or interference-control details. Future work should use prespecified and fully documented microbiological and endotoxin methods, sterility culture when relevant, batch-level documentation, and clinically linked adverse-event surveillance.

Relevance to Adinizer-processed MFAT literature

Previously published Adinizer-related Cureus case reports from this group have focused on clinical applications in osteonecrosis of the femoral head, degenerative lumbar spondylolisthesis, cervical myelopathy, and knee osteoarthritis [7-10]. The present technical note differs by focusing on preliminary laboratory observations of the mechanically processed product using residual adipose tissue. A recent independent multi-device comparison reported that Adinizer-processed nanofat had the highest proportion of adipose-derived stromal/stem cells among eight commercially available preparation systems and achieved the highest overall biological quality score [18]. That investigation evaluated nanofat using its own processing and analytical framework rather than the enzymatically liberated refined MFAT examined here, and it did not report the same mycoplasma or endotoxin outcomes. It therefore provides relevant external context but cannot directly validate or be quantitatively compared with the present findings. Importantly, the present observations provide Adinizer-specific donor-level evidence that viable cells and a laboratory-defined ASC-like fraction remain recoverable after the described mechanical processing and subsequent analytical enzymatic liberation. Given the small sample size and incomplete flow-cytometric documentation, these findings do not establish device-specific cellular benchmarks and should not be used for comparative evaluation with other MFAT preparation systems. Their primary value is to provide preliminary product-characterization data, generate hypotheses, and inform the design of future prospective studies with larger donor cohorts and complete data documentation.

Limitations

This technical note has several important limitations. First, only three donors were included, all were female, and all had BMI values in the obese range; consequently, the observations are descriptive, not generalizable, and cannot be extrapolated to normal-weight or overweight individuals. Second, the retrospective design precluded acquisition of additional markers and functional assays. Third, the compiled flow-cytometry report provided summary percentages and a schematic analytical sequence but did not include raw FCS files, event-level plots, exact gate thresholds, denominator definitions, compensation matrices, staining controls, viable-cell gating details, or isotype/FMO controls. The flow-cytometric fractions reported here should not be interpreted as quantitative evidence of specific cellular subsets for comparative device evaluation or as definitive immunophenotypic characterization. They are descriptive observations derived from a single external laboratory's analytical scheme and cannot be independently reproduced or reanalyzed from the available dataset; prospective validation with fully documented flow-cytometry protocols and raw data deposition is required. Fourth, the endothelial-cell category was not reportable. Fifth, the mycoplasma and endotoxin results were reported as qualitative or binary pass/fail outcomes based on the testing laboratory's internal criteria. Because the assay platforms, analytical sensitivities, positive/negative controls, and interference-testing details were not documented in the source records, these findings do not constitute a comprehensive quality-control release profile and should not be interpreted as establishing sterility, absence of microbial contamination, or product safety. Sixth, no comparator processing device was included. Seventh, cell counting and viability assessment were performed in duplicate as technical replicate measurements from the same analytical preparation and run; these duplicates did not represent independent MFAT processing runs and therefore do not quantify processing reproducibility or between-run analytical variability. Eighth, the 3-mL analytical aliquot was obtained from the final refined MFAT preparation without additional homogenization after completion of mechanical processing. Given the inherent heterogeneity of MFAT, sampling variability may have influenced the measured cellular yield and composition, and the results may not fully reflect the characteristics of the entire product. Ninth, the reported counts represent cells recovered after a specific enzymatic-digestion, filtration, and resuspension protocol; they should not be interpreted as the absolute cellular content of intact MFAT or as the clinical cell dose delivered. Finally, donor-to-donor variation may have been influenced by factors not captured in the dataset, including harvest site, tissue quality, collection technique, and handling conditions. These limitations require that the findings be interpreted as preliminary, descriptive, and hypothesis-generating observations.

## Conclusions

This technical note provides preliminary evidence that viable cells, including a laboratory-defined ASC-like fraction, can be recovered after enzymatic liberation of Adinizer-processed MFAT from three residual adipose tissue specimens. Recovered cell yield varied substantially among donors, and no consistent monotonic pattern between BMI and recovered cell yield was observed within the restricted obese BMI range represented by these donors. The presence of an ASC-like fraction suggests preservation of a stromal/progenitor-compatible cellular component after mechanical processing; however, the limited marker panel and absence of functional assays preclude conclusions regarding definitive mesenchymal stromal/stem-cell identity or functional stemness. Mycoplasma testing was reported as negative in all samples, and all endotoxin results met the testing laboratory’s acceptance criterion of <1 EU/mL; these findings should be interpreted as limited contamination screening results rather than evidence of sterility or product safety. Larger prospective studies using complete flow-cytometric documentation, broader marker panels, and functional assays are required to determine the biological characteristics and potential functional relevance of the cellular populations retained in Adinizer-processed MFAT.
